# Supplementary material for: A Novel Cu2+ Quantitative Detection Nucleic Acid Biosensors Based on DNAzyme and “Blocker” Beacon
Source: Foods. 2023 Apr 3;12(7):1504. doi: 10.3390/foods12071504 (PMC10094606; doi:10.3390/foods12071504)
Supplement: Supplementary file 1 [file foods-12-01504-s001.zip › foods-2271967-supplementary.pdf]

Supplementary Materials

A novel Cu^{2+} quantitative detection nucleic acid biosensors based on DNAzyme and “blocker” beacon

Table S1 Absorbance: Optimized of copper ion cutting conditions (Figure 2C Original data)

	Data 1	Data 2	Data 3
**1: 1.5 M NaCl, 50 mM HEPES, pH 7.0;**	0.178	0.184	0.181
**2: HEPES 100 mM, pH 7.0, 0.008% (V/V) Trition X-100 and 400 mM NaCl**	0.191	0.194	0.198
**3: Ultrapure water**	0.451	0.444	0.431

Table S2 Absorbance: Optimization of chromogenic ionic conditions (Figure 2D Original data)

	Data 1	Data 2	Data 3
**1: 1.5 M NaCl, 50 mM HEPES, pH 7.0;**	0.351	0.343	0.349
**2: HEPES 100 mM, pH 7.0, 0.008% (V/V) Trition X-100 and 400 mM NaCl**	0.553	0.553	0.551
**3: 10 mM Tris-HCl pH 7.4, 10 mM KCl, 100 mM NaCl, 0.002% (V/V) TritonX-100;3: Ultrapure water**	0.143	0.145	0.141
**4: 10 mM Tris-HCl (pH 8.0), 0.002% (V/V) Tri-tonX-100, 1.6 mM KCl, 0.8 mM $MgCl_2$;**	0.231	0.227	0.224
**5: 50 mM NaCl ,10 mM Tris-HCl (pH 7.9) , 10 mM DTT, 10 mM $MgCl_2$;**	0.065	0.061	0.064
**6: Ultrapure water**	0.187	0.194	0.191

Table S3 Absorbance: Optimization of the concentration of H_2O_2 (Figure 2E Original data)

**Concentrations of H_2O_2**	**Data 1**	**Data 2**	**Data 3**
**0.25 mM**	0.35	0.324	0.345
**1 mM**	0.55	0.532	0.543
**1.5 mM**	0.425	0.412	0.418
**2.5 mM**	0.275	0.271	0.264
**5 mM**	0.25	0.234	0.245

Table S4 Absorbance: Optimization of the ratio of Cu-Enz and Cu-Su (Figure 2F Original data)

The ratio of Cu-Enz to Cu-Sub	Data 1	Data 2	Data 3
**1:1**	0.35	0.341	0.345
**1:3**	0.347	0.341	0.341
**1:5**	0.334	0.321	0.317
**1:10**	0.311	0.314	0.315
**1:15**	0.301	0.304	0.3

Table S5 Absorbance (λ = 419 nm) under different Cu^{2+} concentrations (Figure 3B, 3C Original data)

The concentrations of Cu^{2+}	Data 1	Data 2	Data 3
**0 Nm**	0.56	0.562	0.563
**31.25 nM**	0.543	0.543	0.543
**62.5 nM**	0.537	0.537	0.537
**125 nM**	0.53	0.53	0.53
**250 nM**	0.507	0.509	0.511
**500 nM**	0.48	0.48	0.48
**1 μM**	0.455	0.45	0.451
**4 μM**	0.445	0.441	0.448
**8 μM**	0.426	0.428	0.425
**12 μM**	0.381	0.384	0.383
**16 μM**	0.324	0.323	0.321
**50 μM**	0.301	0.304	0.306
**100 μM**	0.276	0.266	0.273

Table S6 Absorbance**:** The change of absorbance (ΔAbs) of the turn-off sensor after adding different metal ions (Figure 4A Original data)

	Data 1	Data 2	Data 3
Cu^{2+}	0.164	0.165	0.161
Fe^{3+}	0.021	0.021	0.018
Fe^{2+}	0.016	0.017	0.016
Al^{3+}	0.015	0.013	0.016
Ca^{2+}	0.024	0.021	0.024
Pb^{2+}	0.016	0.016	0.014
Mn^{2+}	0.02	0.021	0.024
Zn^{2+}	0.022	0.021	0.021
Li^{+}	0.021	0.02	0.021
Mg^{2+}	0.024	0.024	0.027

Table S7 Determination of $Cu2+$ in four different water sample using the biosensor and ICP-MS. (Table2 Original data)

		This method				ICP-MS			
	Concentrations (μM)	Data 1	Data 2	Data 3	RSD	Data 4	Data 5	Data 6	RSD
Dianchi lake	C1	0.208	0.227	0.225	4.75	0.225	0.227	0.232	2.33
	C2	0.106	0.098	0.096	5.29	0.102	0.098	0.1	2.97
	C3	0.329	0.304	0.309	4.21	0.316	0.315	0.32	1.24
Cuihu lake	C1	0.170	0.174	0.193	6.86	0.182	0.187	0.183	2.15
	C2	0.102	0.105	0.093	6.24	0.102	0.098	0.100	2.97
	C3	0.265	0.270	0.290	4.84	0.283	0.279	0.279	1.23

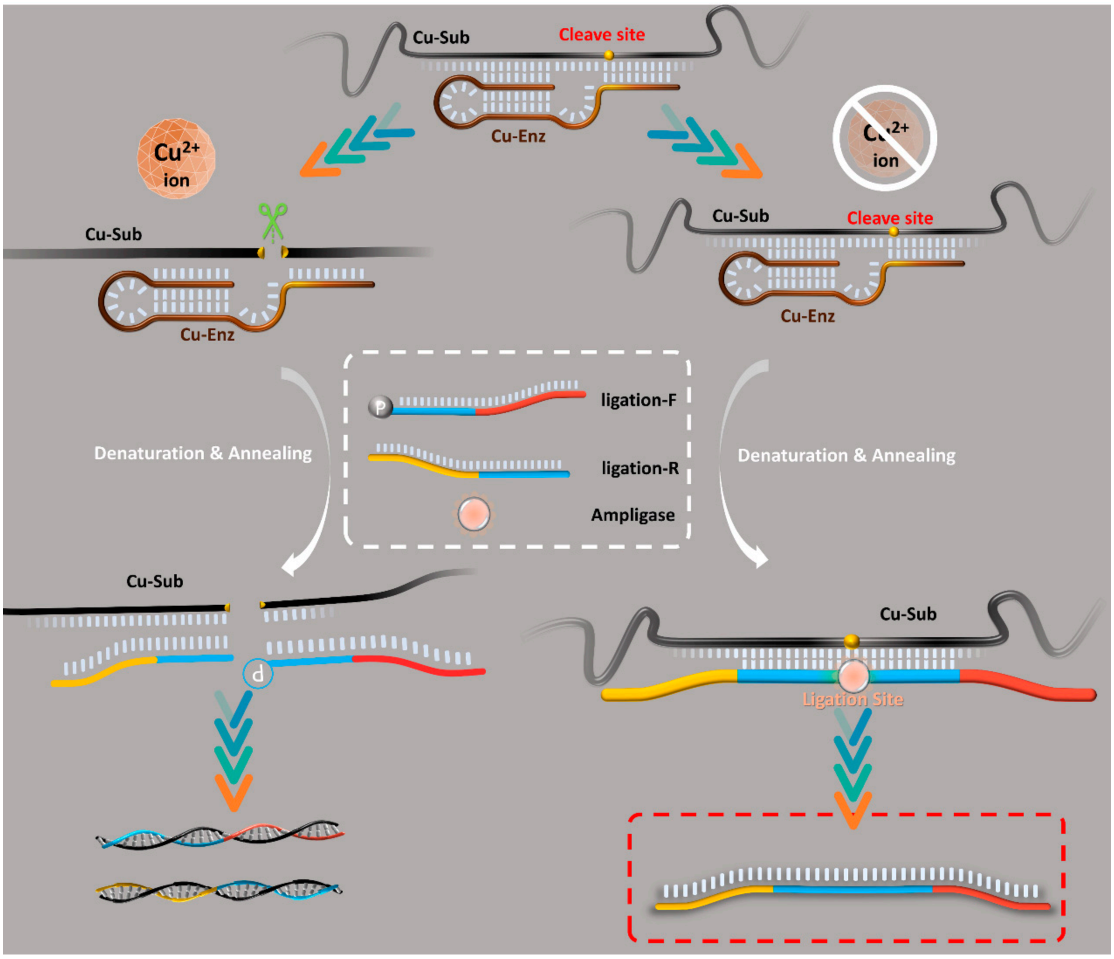

Figure S1: The Original size of Scheme1A

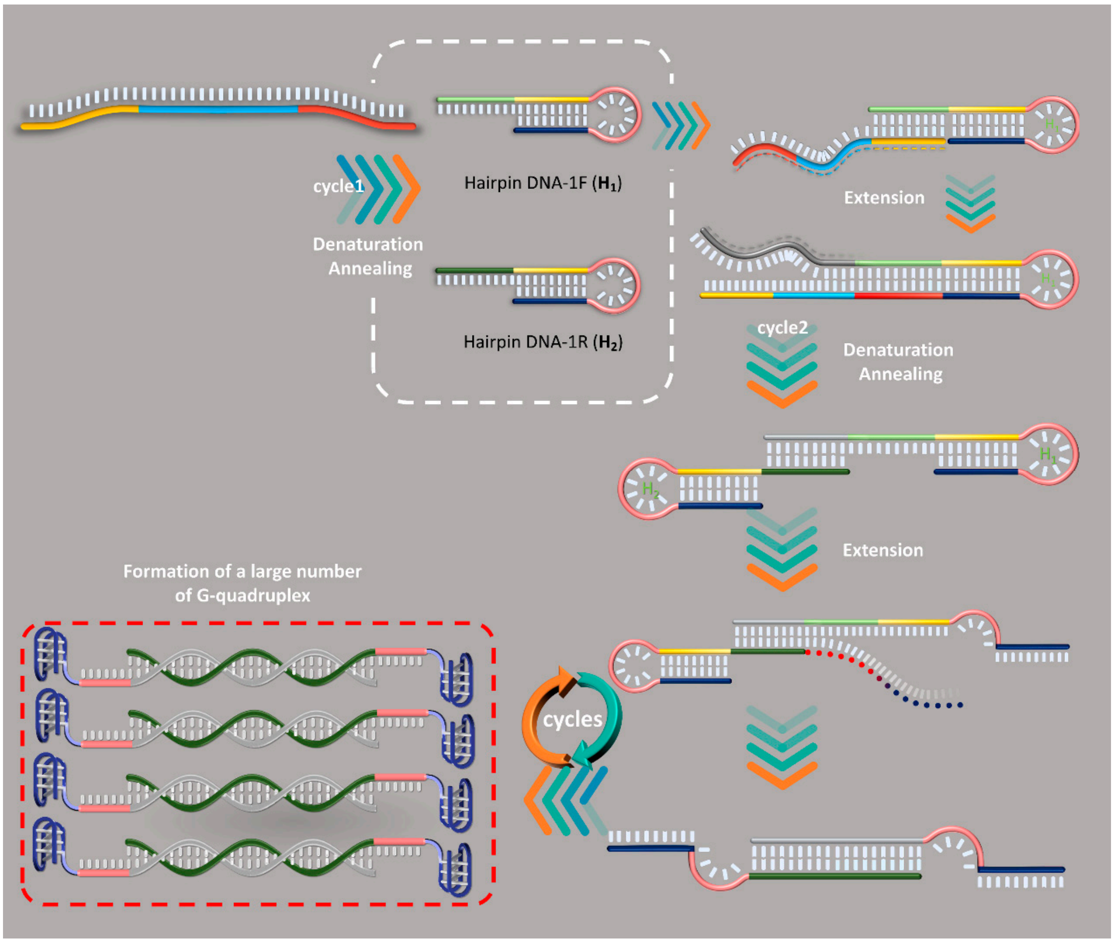

Figure S2: The Original size of Scheme1B

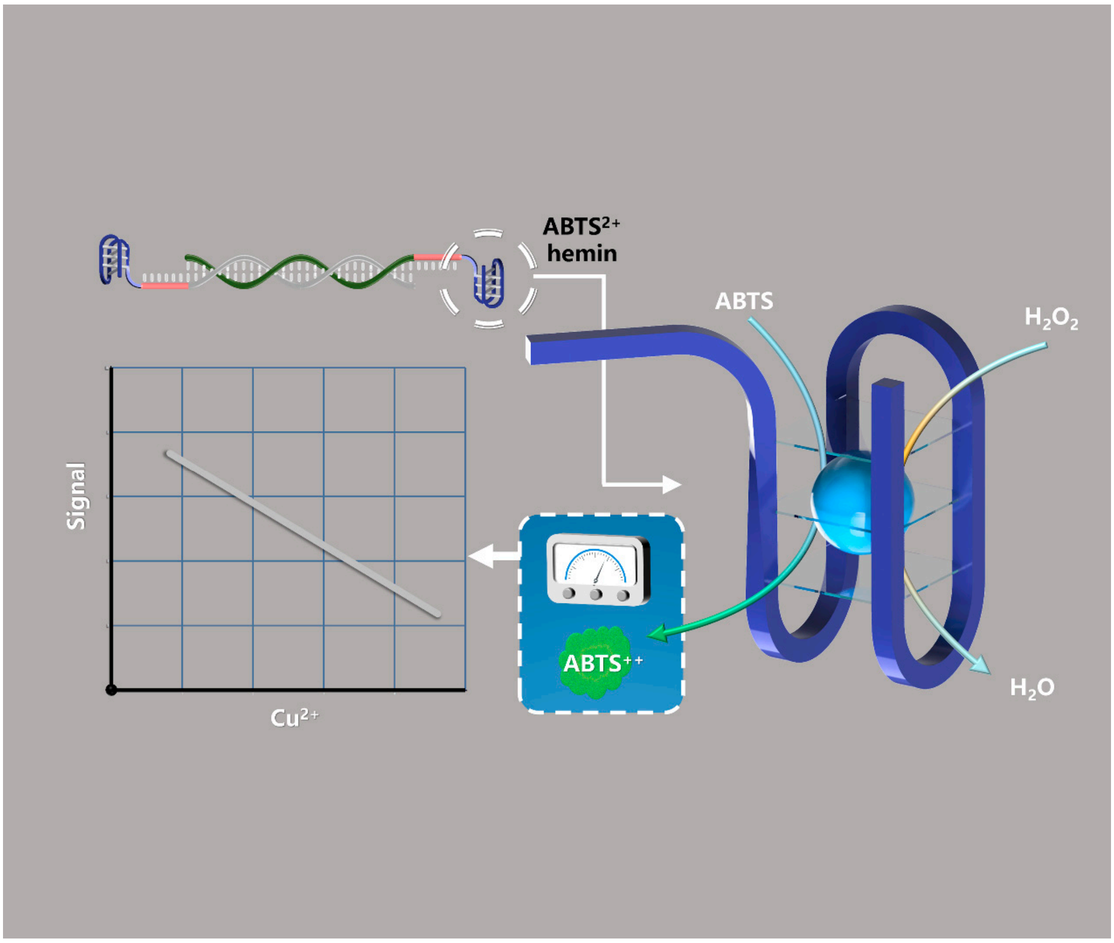

Figure S3: The Original size of Scheme1C
